# Fickian-Based Empirical Approach for Diffusivity Determination in Hollow Alginate-Based Microfibers Using 2D Fluorescence Microscopy and Comparison with Theoretical Predictions

**DOI:** 10.3390/ma7127670

**Published:** 2014-12-01

**Authors:** Maryam Mobed-Miremadi, Sabra Djomehri, Mallika Keralapura, Melanie McNeil

**Affiliations:** 1 Department of Bioengineering, Santa Clara University, Santa Clara, CA 95053-0583, USA; 2 Preventive & Restorative Dental Sciences, University of California San Francisco, San Francisco, CA 94143, USA; E-Mail: sabra.djomehri@ucsf.edu; 3 U-systems, A GE Healthcare Company, Sunnyvale, CA 94085, USA; E-Mail: Mallika@keralapura.com; 4 Department of Biomedical, Chemical and Materials Engineering, San Jose State University, San Jose, CA 95192-0082, USA; E-Mail: melanie.mcneil@sjsu.edu

**Keywords:** alginate, microfiber, diffusivity, modeling, 2D fluorescence, spectrophotometry

## Abstract

Hollow alginate microfibers (*od* = 1.3 mm, *id* = 0.9 mm, *th* = 400 µm, *L* = 3.5 cm) comprised of 2% (*w*/*v*) medium molecular weight alginate cross-linked with 0.9 M CaCl_2_ were fabricated to model outward diffusion capture by 2D fluorescent microscopy. A two-fold comparison of diffusivity determination based on real-time diffusion of Fluorescein isothiocyanate molecular weight (FITC MW) markers was conducted using a proposed Fickian-based approach in conjunction with a previously established numerical model developed based on spectrophotometric data. Computed empirical/numerical (*D*_empiricial_/*D*_numerical_) diffusivities characterized by small standard deviations for the 4-, 70- and 500-kDa markers expressed in m^2^/s are (1.06 × 10^−9^ ± 1.96 × 10^−10^)/(2.03 × 10^−11^), (5.89 × 10^−11^ ± 2.83 × 10^−12^)/(4.6 × 10^−12^) and (4.89 × 10^−12^ ± 3.94 × 10^−13^)/(1.27 × 10^−12^), respectively, with the discrimination between the computation techniques narrowing down as a function of MW. The use of the numerical approach is recommended for fluorescence-based measurements as the standard computational method for effective diffusivity determination until capture rates (minimum 12 fps for the 4-kDa marker) and the use of linear instead of polynomial interpolating functions to model temporal intensity gradients have been proven to minimize the extent of systematic errors associated with the proposed empirical method.

## 1. Introduction

Cross-linked hydrogel-based bio-membranes conferred with selective diffusivities and mechanical properties have been used since the inception of cell-based therapies and implantation [1], with the most common hydrogel biopolymer used being alginate [2,3,4,5]. Due to the versatility of applications, diffusivity quantification across alginate bio-membranes of various geometric shapes and sizes differing by nature and extent of cross-linking and coatings has been extensively researched [6,7,8,9,10,11,12,13]. In conjunction, indirect diffusivity estimations using pore size characterization by various scanning probe microscopy (SPM) methods have been documented [14,15,16]. Other methods include spectrophotometry [17,18,19,20], size exclusion chromatography [8,21], mechanical release tests [22,23] and fluorescence microscopy [23,24,25]. Regardless of the diffusivity characterization method, the following are a sub-set of factors affecting cross-link homogeneity, size and density, gel isotropy and, thus, membrane diffusivity [13,19,20]: (1) the β-d-mannuronic (M) to α-l-guluronic (G) ratio, the distribution of each monomer within the chain, and alginate molecular weight (MW); (2) the kinetics of gelation, specifically external gelation (chelation) *vs.* internal gelation.

Spectrophotometry and fluorescence microscopy are non-destructive as compared to SPM methods requiring sample immobilization, morphology modifications and cross-sectioning, introducing artifacts. The advantage of fluorescence over spectrophotometry is the measurement accuracy at lower detection limits [26,27], which can be a source of error when validating numerical or analytical models derived from Fick’s laws. The diffusivity of multiple compounds across hydrogels using transient Fickian-based models has been modeled with the equilibrium concentration as a boundary condition [6,18,25]; thus, the validity of the empirical model hinges on the lower limit of detection. Data smoothing is often a recourse to eliminate the noise from the oscillatory equilibrium concentration. In these cases, it is difficult to deconvolute the sensitivity of the measurement technique from the predictive accuracy of the models. Another advantage of fluorescence microscopy over spectrophotometry is the ability to directly see the qualitative effects of diffusion through a hydrogel membrane, while simultaneously acquiring large amounts of real-time quantitative measurements.

In this paper, outward flux measurements (*J*) captured by fluorescence microscopy have been correlated to the change in concentration (*dC*) in the radial direction (*dr*) across a hollow fiber wall in order to determine the effective diffusivity (*D_e_*). According to Fick’s first law, the instantaneous flux is given by Equation (1), where *M* is the amount of diffusing solute in volume *V* and unit area *A* available for mass transfer of the solute:

(1) $$J=\frac{dM}{dt}=V\frac{dC}{dt}=-AD_{e}\frac{dC}{dr}$$

Fick’s second law in cylindrical coordinates describing the transient solute radial diffusion as a function of diffusivity (*D_e_*) across the membrane is given by Equation (2):

(2) $$\frac{\partial C_{m}}{\partial t}=\frac{1}{r}\frac{\partial }{\partial r}\left(D_{e}r\frac{\partial C_{m}}{\partial r}\right)$$

The Renkin–Curry equation used to estimate free solution diffusivity (*D*) is given by Equation (3), and the Stokes’ radius (*a*) can be calculated from the Stokes–Einstein equation (Equation (4)), where μ is the solution viscosity [28]:

(3) $$D=1.013\times 10^{-4}{\left(MW\right)}^{-0.46}$$

(4) $$a=\left(\frac{RT}{6\pi \mu DN_{A}}\right)$$

The ratio of membrane to free solution diffusivity is a product of the steric exclusion/partition coefficient (*K*) and hydrodynamic effect (ω*_r_*), given by Equation (5), where *r* is the membrane pore size [24]:

(5) $$\frac{D_{m}}{D}={\left[1-\left(\frac{a}{r}\right)\right]}^{2}\left[1-2.1\left(\frac{a}{r}\right)+2.09{\left(\frac{a}{r}\right)}^{3}-0.95{\left(\frac{a}{r}\right)}^{5}\right]=K\left(\frac{a}{r}\right)\times \omega_{r}\left(\frac{a}{r}\right)$$

If the solute freely diffuses between the membrane pores and the bulk solution, then the value of *K* can be approximated as unity.

Assuming a porosity (ϵ = *A_p_*/*A*) of one, the correlation between *D_e_*, *D* and *D_m_* is given by Equation (6), where τ*o* is membrane tortuosity [24]:

(6) $$D_{e}=\frac{D}{\tau }K\omega_{r}=\frac{D_{m}}{\tau o}$$

This is an extension of previous research where diffusion across porous hollow stents comprised of cross-linked alginate/chitosan with tunable permeability were simulated using a cylindrical model based on Fick’s diffusion equations [18]. Experimental parameters were set to match the initial condition and boundary conditions based on solute equilibration data obtained spectrophotometrically for the inward diffusion of solutes. In this work, the use of a proposed empirical approach for the determination of membrane diffusivity (*D_m_*) based on Fick’s laws, consisting of the superimposition of finite radial and temporal intensities obtained using real-time 2D fluorescence microscopy, will be explored. The range of empirical diffusivity values for several MW markers *D_e_* will be compared to the theoretical diffusivity computed using the above-mentioned numerical model with flipped boundary conditions [18]. Using the numerical diffusivities as the benchmark, biomedical researchers can simulate and validate radial micro-measurements related to intra-membrane fluxes across porous fiber-based engineered tissues for tuning membrane composition and modeling metabolic trends [29,30,31,32]. This methodology could be extended to quantify transport across emerging stimuli-responsive macro-/micro-fibers and nanocomposites in wound healing [33,34,35,36].

## 2. Results and Discussion

### 2.1. Results

#### 2.1.1. Hollow Fiber Fabrication

**Figure 1:**
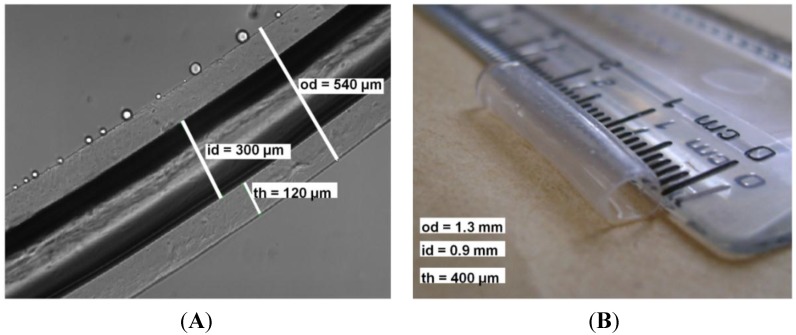
Hollow alginate microfiber fabricated with a metallic rod with a diameter of (**A**) 300 µm and (**B**) 0.9 mm. Figure 1A reproduced with permission from [18].

Shown in Figure 1A is a micrograph of a hollow fiber fabricated from 2% (*w*/*v*) medium molecular weight alginate cross-linked with 0.9 M CaCl_2_ for 60 min using a 0.3-mm metallic rod (*od* = 540 μm, *id* = 300 μm, *th* = 120 µm, *L* = 3.5 cm). Shown in Figure 1B is a hollow fiber fabricated using a 1-mm metallic rod (*od* = 1.3 mm, *id* = 0.9 mm, *th* = 400 µm, *L* = 3.5 cm). Throughout the paper, diffusion will be modeled through a wall thickness of 400 µm.

#### 2.1.2. Diffusion Imaging Using Fluorescence

**Figure 2:**
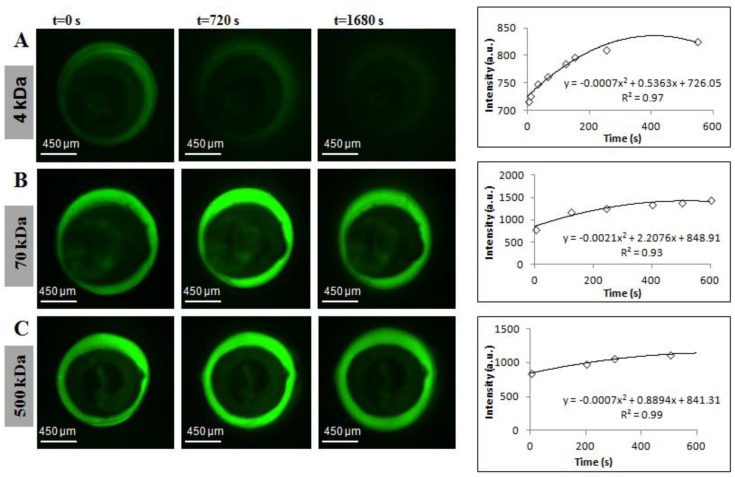
Fluorescence diffusion profiles of FITC-dextrans from hollow alginate microfibers and corresponding intensity changes in the bulk solution. Molecular weights used to perform empirical diffusion experiments were: (**A**) 4 kDa; (**B**) 70 kDa; and (**C**) 500 kDa.

Outward diffusion of all MW fluorescent markers is captured by diffusion imaging, as shown in Figure 2 and Movie 1 (see Supplementary). Bright green regions in Figure 2B,C demonstrate qualitatively the slow movement of 70-kDa and 500-kDa dextrans through the alginate membrane, whereas the faintly green regions in Figure 2A demonstrate rapid diffusion of 4-kDa dextran over the course of 28 min. Quantitative analysis over a time course of 10 min, comprised of corresponding intensity *vs.* time plots fitted to parabolic equations, was implemented in the numerical diffusion model using MATLAB R2013a (Natick, MA, USA).

#### 2.1.3. Modeling Summary of Diffusivities by Empirical and Numerical Approaches Using Fluorescence Microscopy

**Figure 3:**
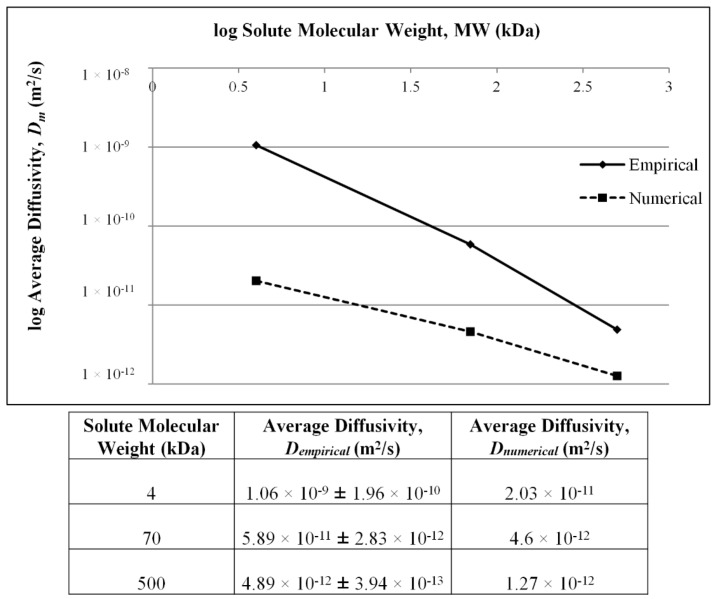
Summary of computed diffusivities for MW markers using the empirical (*D*_empirical_) and numerical methods (*D*_numerical_).

A comparative summary of diffusivities computed using the proposed empirical (*D*_empirical_) and numerical methods (*D*_numerical_) is presented in Figure 3. For both methods, data acquisition for fluorescent solutes was performed in triplicate, from which the average diffusivities were calculated. The largest discrepancy, up to two orders of magnitude between the two methods, is observed for the 4-kDa marker, with the gap between the detection techniques narrowing down as a function of solute MW. Although discrepancies in magnitude are observed, the standard deviation for each computed value is a fraction of the average value indicative of consistent sample behavior and robust modeling.

As theoretically expected, findings using both methods converge to a negative correlation between diffusivity and MW, as shown in Figure 3. Effective diffusivities are inversely proportional to the respective Stokes radii of the solutes independent of computational methods according to the Renkin–Curry theory for cylindrical pores [28].

### 2.2. Discussion

#### 2.2.1. Impact of Assumptions on Diffusivity Estimation

Regardless of the diffusivity computation method, the assumptions listed below are a systematic source of error. Throughout the study, the partition coefficient (*K*) has been assumed to be one. According to Equation (5), *K* should decrease as a function of MW. Since the membrane pore size *r* is unknown, the exact value of *K* cannot be computed, and *D_e_* values have not been normalized by MW. Knowledge of *r* would also enable the calculation of the hydrodynamic drag (ω*_r_*) [37].

Other assumptions are the values of porosity (ϵ) and tortuosity (τ*o*) taken as unity, resulting in an overestimate and underestimate of *D_e_*, respectively.

In light of the above assumptions, in this study, *D_m_* and *D_e_* will be interchangeably used, and the value of *D_e_* should always be lower than *D*.

#### 2.2.2. Effect of MW Probe Size on Diffusivity

**Figure 4:**
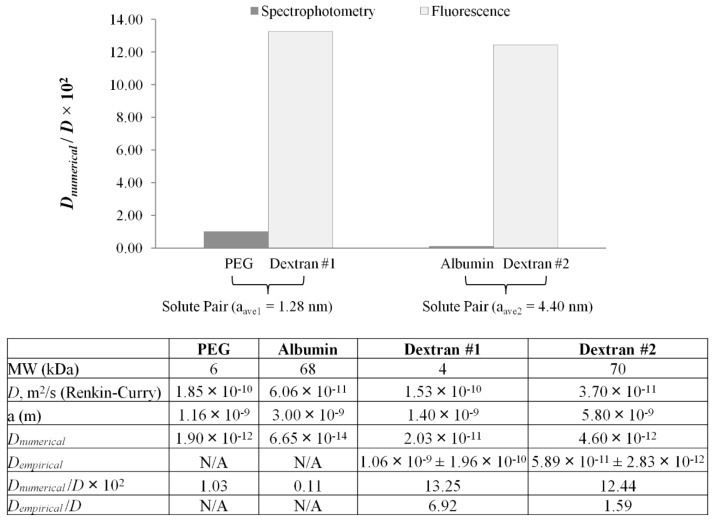
Comparison of numerical diffusivity ratios (*D*_numerical_/*D*) between spectrophotometry and 2D fluorescence. Average stokes radii for each solute pair are as follows: *a*_ave1_ = 1.28 nm for polyethylene glycol (PEG)/dextran #1, and *a*_ave2_ = 4.40 nm for albumin/dextran #2. Numerically-computed diffusivity (*D*_numerical_) for PEG and dextrans previously reported in [16].

Across measurement techniques, reported values of pore size for alginate membranes range between 3.6 nm and 10.9 μm [12,15,16,23,25,37,38,39,40,41,42,43,44,45,46]. For the external gelation fabrication method, the molecular weight cutoff (MWCO) of the membrane has been placed between 60 and 70 kDa (≈3–6 nm) by multiple sources [7,12,20,25,37,47]. It is essential to distinguish pure diffusion from surface erosion, the latter being a combination of hydrodynamic drag and membrane swelling [13,48]. As shown in Figure 3 and Figure 4, effective diffusivity decreases as a function of the MW of the probe. The definition of “lack of permeability” hinges on whether the MWCO of the 60–70-kDa marker is a valid hypothesis or not. If proven true, membrane erosion has been measured for the 70-kDa and 500-kDa markers. The chronological diffusion profiles in Figure 2 for these markers support the validity of the hypothesis.

The closest study in terms of design to the current effort is the use of fluorescence recovery after the photobleaching (FRAP) technique for measuring the diffusion of 10-kDa, 70-kDa and 500-kDa markers across externally-gelled alginate membranes prepared by chelating a 1.8% (*w*/*w*) alginate solution comprised of 70% G groups by the addition of 0.5 M CaCl_2_ [23]. The (*D_m_*/*D*) ratios reported are 0.8, 0.5 and 0.2, for the 10-kDa, 70-kDa and 500-kDa probes, respectively. Assuming that the 4-kDa and 10-kDa marker have approximately the same diffusivity, by multiplying the above ratios by the Stokes’ Einstein diffusivity *D* and dividing the resultant by *D*_numerical_, a set of ratios can be obtained to compare the findings. Membrane diffusivities obtained using FRAP are 6.0-, 4.0- and 2.3-times larger than the numerical diffusivities for the 4-kDa, 70-kDa and 500-kDa markers, respectively. In turn, empirical diffusivities are 8.7-, 3.2- and 1.7-times larger than the FRAP study diffusivities for the 4-kDa, 70-kDa and 500-kDa markers, respectively. This reported discrepancy between *D*_numerical_ values from the two studies could be attributed to a number of factors, namely alginate composition, concentration, fabrication method, morphology and cross-linker concentrations and detection techniques. In the case of the latter, it is also stated that the membranes is permeable to 70 kDa.

#### 2.2.3. Modeling Summary of Diffusivities by Empirical and Numerical Approaches Using Fluorescence Microscopy

A possible source of measurement-related systematic error is the upper limit for the capture rate governed by equipment capability, leading to a lack of sampling and, thus, an inaccurate reconstruction of spatial and temporal intensity gradients. This limitation can be mitigated by image capture capabilities exceeding 12 fps. In parallel, possible sources of systematic processing errors are: (1) the concentration inside the membrane obtained by averaging five intensities at a given radial position not being uniformly-distributed, contributing to inaccuracies in intensity measurements at the membrane interface (for *C_m_*_0_ and *C_m_*); (2) although the dimensionless initial condition and boundary conditions were created (Equation (14b–d)) for the numerical model, the theoretical assumption is for a solid infinite cylinder with an impermeable core, whereas in reality, a finite hollow cylinder of length 3.5 mm was used; and (3) applicable to the empirical method only, the use of a polynomial model to measure the temporal intensity gradient (*dI_b_*/*dt*) as illustrated in Figure 2.

Proposed respective contingencies to reduce processing errors are as follows: (1) finite element analysis methods can be used for the estimation of membrane intensity distribution [49]; (2) 3D fluorescence imaging can be used to tune the boundary conditions [50] and to verify whether radial gradients are constant in the z direction; and (3) piecewise linear or polynomial interpolation is preferred over creating a single interpolating polynomial function that passes through each point to reduce the error of the fit [51]. The error of the fit can be, in turn, reduced by higher capture rates.

#### 2.2.4. Comparison of Spectrophotometry and 2D Fluorescence Microscopy

Previously, spectrophotometric measurements were used to determine diffusivity values across alginate-based microfibers identical in morphology and composition using the above-mentioned numerical diffusion model and non-fluorescent solutes; namely creatinine, PEG-6000 (polyethylene glycol) and albumin [18]. It could be hypothesized that identical solutes were not used due to the unknown effects of set-up and photobleaching associated with UV-Vis spectrophotometry [52,53]. PEG-6000 was chosen as the model intermediate MW molecule simulating the behavior of toxins during hemoperfusion, a treatment for which the use of alginate fibers instead of the use of cellulose nitrate and polyamide has been explored [54]. Albumin was chosen as a model high MW solute reported to be at the molecular weight cutoff of cross-linked alginate membrane in spherical configuration [1,7,18,25]. Pairwise comparisons of PEG-6000/FITC 4 kDa and albumin/FITC (Fluorescein isothiocyanate) 70 kDa were conducted, since the paired solutes have approximately the same Stokes radius. Shown in Figure 4 is a comparison of numerically-computed diffusion ratios (*D*_numerical_/*D*) determined by both spectrophotometric and fluorescence techniques. The average Stokes radius of each solute pair has been used (*a*_ave1_ = 1.28 nm for PEG-6000/FITC 4 kDa and *a*_ave2_ = 4.40 nm for albumin/FITC 70 kDa). This was accomplished by fitting spectrophotometric and fluorescence data to the numerical model to attain *D*_numerical_. The free solution diffusivity (*D*) and Stokes radii were determined using Equations (3) and (4), respectively. The ratio (*D_e_*/*D*) referred to in this study as the diffusivity ratio is an indicator of resistance to diffusion. The smaller the ratio, the less permeable the membrane is to a specific solute, whereas values of *D_e_*/*D* ≥ 1 are indicative of either membrane disintegration, an artifact of image capture and/or inadequate data processing.

For the same molecular size, spectrophotometric measurements yield significantly lower diffusion ratios than fluorescence microscopy. Aside from the detection method, differences can be explained by solute-membrane interactions. The discrepancy between albumin and 70-kDa dextran arise from electrostatic interactions at the membrane; namely, albumin with an isoelectric point (PI) of 4.7 [55], being negatively charged at pH = 7.4. Electrostatic interactions also occur with PEG at the membrane, a bipolar molecule with both hydrophilic and hydrophobic groups (PI = 6.2) [56]. For PEG and albumin, charge-associated effects interfere with the true diffusive ability of the solute, and thus, the diffusion coefficient can be significantly underestimated. As a result, this method indicates that the diffusivity across the alginate membrane could be larger than previously observed with spectrophotometry. In fluorescence microscopy, the diffusing solutes are FITC-dextrans, which are neutral and hydrophilic molecular weight markers. The higher diffusivity range determined for fluorescent dextrans could represent a more accurate result compared to spectrophotometry, due to the pure diffusive behavior occurring without electrostatic effects.

In addition, diffusivity ratios across measurement techniques are significantly smaller than unity, except for the case of 4 kDa (*D*_empirical_/*D* = 6.92) and 70 kDa (*D*_empirical_/*D* = 1.59). Since no membrane disintegration was observed and the image capture scheme is common to both empirical and numerical methods, the source of error could be narrowed down to the empirical modeling process. One such source could be the use of a polynomial model to measure the temporal intensity gradient (*dI_b_*/*dt*) elaborated upon in the explanation of the processing errors associated with fluorescent measurements.

## 3. Experimental Section 

### 3.1. Materials

All chemicals used to make the capsules were purchased from Sigma Aldrich (St. Louis, MO, USA): low molecular weight sodium-alginate (LV) (A0682, *M_v_* *=* 12–80 kDa, M/G ratio 1.6), medium molecular weight alginate (A2033, μ > 2000 cP, *M_v_* = 900–1000 kDa, M/G ratio 1.6) and fluorescein isothiocyanate dextran markers, abbreviated as FITC-dextran markers (46947, FD70S, FD4).

### 3.2. Methods

#### 3.2.1. Hollow Fiber Fabrication

Hollow fibers were fabricated by a mold casting external gelation approach similar to that conducted by Barralet, *et al.* [57]. A metallic rod was used to produce hollow fibers by submerging them into a 2% (*w*/*v*) medium viscosity (MV) alginate mixture, which has an equivalent dynamic viscosity to a 3% (*w*/*v*) MV sterilized solution. The diameter of the hollow fiber is determined by the diameter of the metallic rod. As a thin layer of alginate coats the rod, it is then submerged into a 0.9 M CaCl_2_ bath for 1 h of cross-linking [18]. Microfibers were subsequently removed from the rod and rinsed twice with NaCl (0.154 M), and the resulting microfibers are shown in Figure 1. The preparation of fluorescent hollow fibers was the same as the protocol for producing hollow alginate fibers, except the starting alginate solution is prepared at a higher concentration than 2% (*w*/*v*) to compensate for the dilution once mixed with fluorescent dextran standards of known concentration for 2 h. The final concentration of FITC-dextrans in the fluorescent alginate mixture for the 4, 70, and 500 kDa were 0.1, 5 and 5 mg/mL, respectively. The mixture was covered in aluminum foil during mixing to eliminate photobleaching. The mixture of alginate and fluorescent dextran was used to fabricate microfibers according to the above-described procedure.

#### 3.2.2. Optical Measurements

Membrane thickness and stent diameter were measured using a Nikon transmission microscope/camera (Nikon Eclipse*Ti-S*, Nikon, Melville, NY, USA) equipped with an Andor Technology Interline (Andor Technology Ltd., Belfast, Northern Ireland).

For fluorescent measurement, the FITC/Acridine Orange filter was chosen from the imaging software (NIS-Elements v.3.2.2, Nikon, Melville, NY, USA) filter selection feature to accommodate the excitation and emission wavelengths of 468 and 520 nm of the FITC molecule. Pixel values in the NIS-Elements software were calibrated to distance, where 1 pixel = 5.09 µm.

#### 3.2.3. Calibration Solutions Preparation

**Figure 5:**
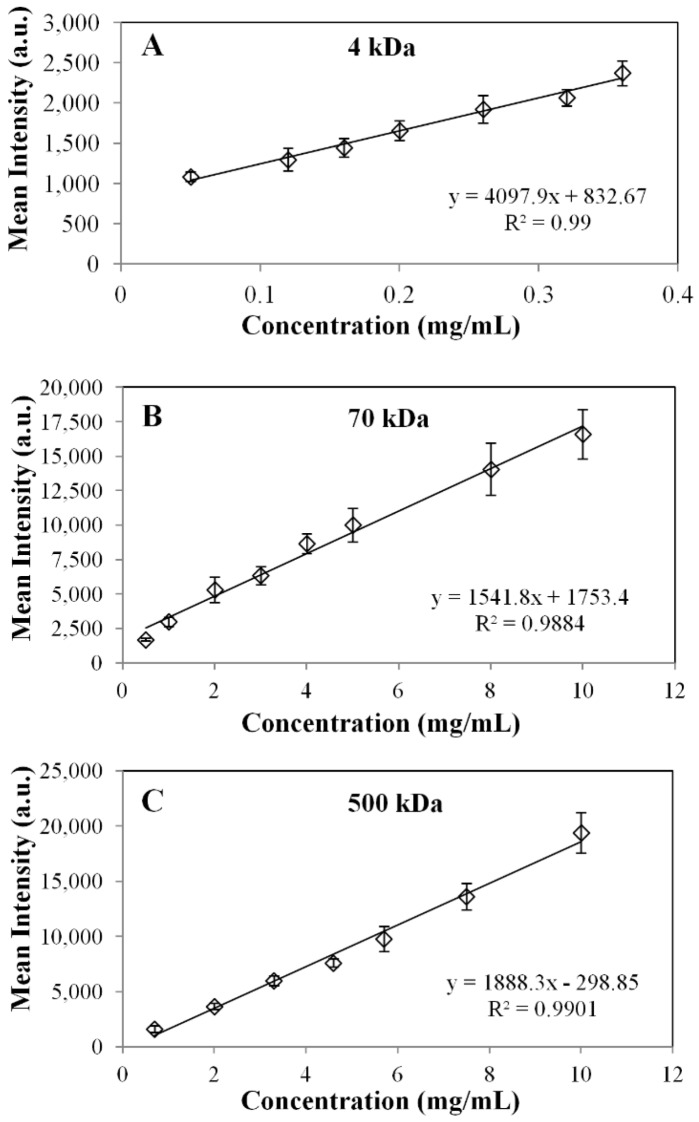
Sample calibrations curve for the FITC MW probes: (**A**) 4 kDa; (**B**) 70 kDa; and (**C**) 500 kDa.

Calibration stock solutions ranging from 0.05 to 10 mg/mL for FITC-dextran MW standards (4, 70 and 500 kDa or stokes radii of 1.4, 5.8 and 14.5 nm) dissolved in 0.154 M NaCl were prepared. In dilute solutions, there is a linear relationship between intensity and the concentration of the fluorescent marker under observation [26]. Hence, calibration and outward diffusion measurements were carried out over this range. Droplets of 5 µL of either 4, 70 or 500 kDa FITC-dextran were arranged on a glass slide. The slide was then placed on the stage of the microscope, and an image was taken for each droplet. The imaging software region of interest (ROI) tool was then used to determine the fluorescent intensity of the entire region. Mean intensity values were delivered as the output. A mean intensity *vs.* known concentration calibration curve weregenerated. Shown in Figure 5A–C are the sample calibration curves for 4-, 70- and 500-kDa FITC-dextrans.

#### 3.2.4. Sample Preparation

**Figure 6:**
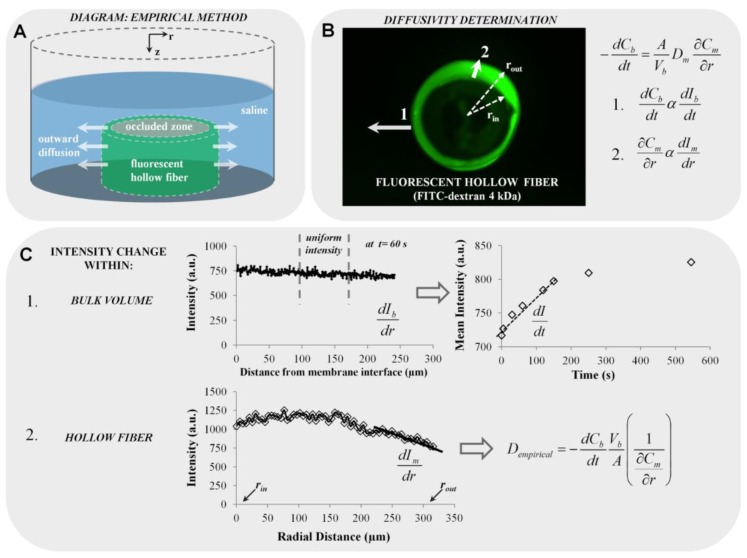
Description of the proposed methodology for the empirical diffusivity determination. (**A**) Diagram of fluorescent hollow fiber situated in a chamber of 1 mL of saline solution simulating outward diffusion (not drawn to scale); (**B**) fluorescent hollow fiber image (e.g., FITC-dextran 4 kDa is shown); (**C**) scheme of fluorescence diffusion measurements with Line Plot 1 representing intensity changes in the bulk volume, *V_b_*, and Line Plot 2 representing intensity changes within the hollow fiber membrane (*r_out_*, outer radius; *r_in_*, inner radius).

The diagram for the experimental setup is shown in Figure 6A, illustrating a fluorescent hollow stent sectioned along its axial direction to a length of *L* = 3.5 mm. A plastic occlusion was placed in the center of the stent to prevent diffusion toward the stent’s center and eliminate diffusion along the longitudinal axis. The complex was fastened to the bottom face of a cylindrical chamber (*L* = 12 mm, *d* = 12 mm). The plastic occlusion also provides a support with which to prop the stent upright. Diffusion is most effectively observed through the radial plane, *r*, which occurs when the longitudinal axis, *z*, of the stent is perpendicular to the stage of the microscope.

#### 3.2.5. Image Capture

Once the wall thickness of the stent was clearly visible, a 1-mL volume of saline solution was injected into the chamber to cover the stent. Images were acquired every 5 s for the first 5 min, every 15 s for the next 10 min and every minute for the last 15 min. Data acquisition was conducted at a maximum setting of 12 fps and 20× magnification. An image sequence was set up for each test case; the rate and quantity of image capture was pre-defined and resulted in an image stack to be acquired after each run was complete. This given image stack was used to determine the change in intensity within the stent wall thickness and in the bulk solution.

#### 3.2.6. Diffusion Modeling by Fluorescence Measurements: Empirical Approach

In modeling outward diffusivity, the mass balance equation (Equation (7)) based on Fick’s first law is used to solve for *D_e_*. A systematic scheme for membrane diffusivity measurements is outlined in Figure 6B:

(7) $$-\frac{dC_{b}}{dt}=\frac{A}{V_{b}}D_{e}\frac{\partial C_{m}}{\partial r}$$

Line profiles were used to export intensity values at different times (solid white Arrows 1 and 2, Figure 6B). Data acquired from Line Profile 1 extended from the membrane interface to approximately 300 µm into the bulk solution, and that for Line Profile 2 extended from *r_in_* (inner radius) to *r_out_* (outer radius). At each time point, line profiles were taken at the same location. Since this wasan outward diffusivity test, the bulk solution was not well-mixed, resulting in slight non-uniformity at the outer wall of the hollow fiber. To account for this, intensity values in *V_b_* were averaged within a quiescent region best representative of uniform intensity, since the diffusion model assumes bulk concentration (*C_b_*) uniformity. Throughout the study, the intensity averaged over 100–175 μm from the wall was taken as *I_b_*. A sample plot is shown at *t* = 60 s (upper plot, Figure 6C). Due to the linearity of the calibration curves, the changes in concentration are proportional to the changes in intensity (Figure 2B), providing the following assumptions:

(8) $$\frac{dC_{b}}{dt}\alpha \frac{dI_{b}}{dt}$$

(9) $$\frac{\partial C_{m}}{\partial r}\alpha \frac{dI_{m}}{dr}$$

Mean intensity values acquired from the bulk solution were plotted against time, and the largest slope (*dI_b_*/*dt*) was determined (e.g., between 0 < *t* < 200 s in the sample plot). The large slope was indicative of the largest solute flux into the bulk solution to generate the maximum diffusivity, *D_m_*. By a similar approach, Line Profile 2 acquired concentration in the membrane (*C_m_*) as intensity values in the fluorescent membrane (*I_m_*), and the slope (*dI_m_*/*dr*) near the membrane wall at *r_out_* was directly determined (lower plot in Figure 6C). The slopes *dI_b_*/*dt* and *dI_m_*/*dr* along with values for the surface area of the hollow fiber and bulk volume (*A* = 1.44 × 10^−5^ m^2^, *V* = 1 mL) were subsequently input into the rearranged diffusion equation (Equation (5)) to acquire the empirically determined diffusivity, *D*_empirical_, as shown in Figure 6C.

#### 3.2.7. Diffusion Modeling: Numerical Approach

**Figure 7:**
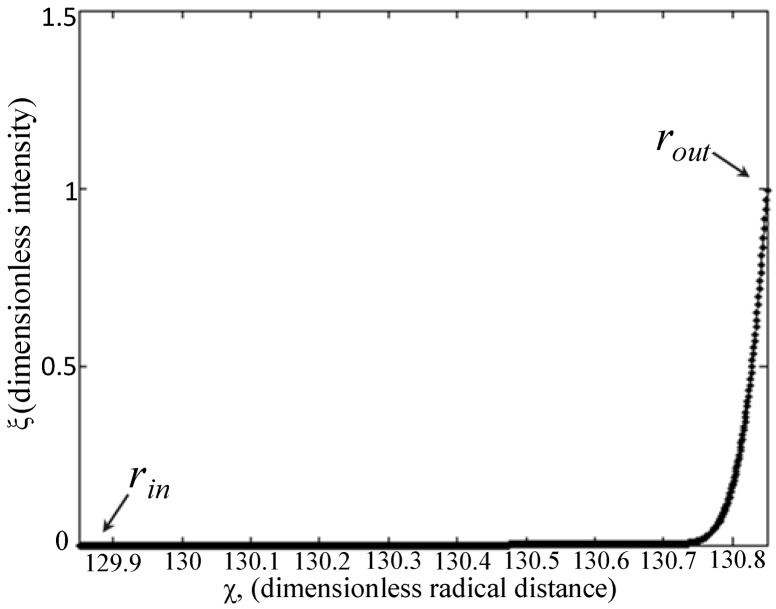
Sample dimensionless diffusion profile of FITC-dextrans, 4, 70 and 500 kDa for the approximation of *D_m_*.

Diffusivities found by the empirical method can be correlated with a previously-established numerical method [18]. The numerical method is a mathematical model for cylindrical diffusion equations of solutes through a microfiber membrane and involves a single partial differential equation and a Neumann boundary condition. The model is a modification of a spherical diffusion model for microspheres [7], with diffusion equations derived by Carslaw and Jaeger [58]. All physical quantities were non-dimensionalized (*i.e.*, *t*, *C*, and *r*):

(10) $$\tau \equiv \frac{D_{e}t}{{\left(r_{out}-r_{in}\right)}^{2}}$$

(11) $$\xi_{m}\equiv \frac{C_{m}-C_{m0}}{C_{b0}-C_{m0}}$$

(12) $$\xi_{b}\equiv \frac{C_{b}-C_{m0}}{C_{b0}-C_{m0}}$$

(13) $$\chi \equiv \frac{r}{r_{out}-r_{in}}$$

where τ, ξ*_m_*, ξ*_b_* and χ are non-dimensionalized time, concentration at the membrane interface (at *r_out_*), concentration in the bulk solution and radial distance, respectively. The constant *C_m_*_0_ is the concentration in the membrane at *r_out_* at *t* = 0 s, and *C_b_*_0_ is the concentration in the bulk solution at *r_out_* at *t* = 0 s. Using Equations (10)–(13), the non-dimensionalized cylindrical PDE was previously in the form of Equation (14a) [18],

(14a) $$\frac{\partial \xi_{m}}{\partial \tau }=\frac{1}{\chi }\frac{\partial }{\partial \chi }\left(\chi \frac{\partial \xi_{m}}{\partial \chi }\right)$$

(14b) $$\xi_{m}\left(\chi ,0\right)=0$$

(14c) $$\frac{d\xi_{m}}{dt}\left(\chi ,\tau \right)=0\;\text{at}\;\chi_{in}=\frac{r_{in}}{r_{out}-r_{in}}$$

(14d) $$-\frac{d\xi_{b}}{d\tau }=2\frac{V_{2}}{V_{b}}\left(1-\frac{r_{in}}{r_{out}}\right)\frac{\partial \xi_{m}}{\partial \chi }\;\text{at}\;\chi_{out}=\frac{r_{out}}{r_{out}-r_{in}}$$

where *V_b_* is the bulk volume and *V*_2_ is the hollow microfiber volume (*V*_2_* =* π*R*_2_^2^*h*); in the case of fluorescence experiments, bulk concentration values were measured as intensities, since concentration is proportional to intensity, as shown by the calibration curve in Figure 5. Intensity profiles in Figure 2A–C for each FITC-dextran were correlated to second order polynomials and input into MATLAB. Both *C_m_*_0_ and *C_b_*_0_ are non-zero constants and measured as intensity values. The initial condition (Equation (14b)) is derived from Equation (11). The non-dimensionalized intensity *vs.* non-dimensionalized radial distance (solution to Equation (14d)) is plotted in Figure 7, where a sample curve for the numerical method of approximation of *D_m_* is illustrated. This approximation is only valid when ξ*_m_* = 1 (Equation (11)), which occurs when an equilibrium concentration has been established in the bulk solution at large values of *t* (*t* ≈ 400 s for 4 kDa FITC-dextran and *t* ≈ 800 s for 70 kDa and 500 kDa FITC-dextran). Note, the value of *C_b_*_0_ was also chosen when equilibrium had been reached. In addition, the statistical accuracy of *D_m_* as solved in MATLAB is dependent on the number of partitions in the PDE solver. The approximation of *D_m_* was found to converge when the number of partitions is ≥1000.

## 4. Conclusions

Diffusion across hollow alginate microfibers (*od* = 1.3 mm, *id* = 0.9 mm, *th* = 400 µm, *L* = 3.5 cm) has been studied using 2D fluorescence microscopy. Effective diffusivities were determined using a proposed Fickian-based empirical approach and a previously established numerical method. For the numerical approach, *D_e_* was estimated to be in units of m^2^/s 1.06 × 10^−9^, 5.89 × 10^−11^ and 4.89 × 10^−12^, for the 4-kDa, 70-kDa and 500-kDa MW probes, respectively. While the membrane was permeable to the 4-kDa marker, transport of the 70-kDa and 500-kDa transport across the membrane were hindered over time proven by real-time fluorescence imaging. Discrepancies up to two orders of magnitude were observed between the numerical and empirical approaches with the discrimination between the predictions narrowing down as a function of diffusing solute MW.

The fluorescence-based findings were compared to spectrophotometric measurements conducted on diffusing solutes with the same order of magnitude of Stokes’ radii. Numerically-computed diffusivities using spectrophotometry measurements were significantly lower than those computed based on fluorescence measurements. Apart from the difference in measurement technique for PEG 6000 and albumin, electrostatic effects interfere with the true diffusive ability of the solute, and thus, the diffusion coefficient can be significantly underestimated.

Future studies using 3D fluorescence microscopy, faster capture rates and the use of linear interpolants should be conducted to reduce the extent of systematic errors for the validation of the empirical approach. Pore size, porosity and membrane tortuosity characterization are also recommended to improve the accuracy of effective diffusivity predictions independent of the mathematical approach, to deconvolute the effect of erosion and pure diffusion at the membrane surface.

## Supplementary Materials

Supplementary materials can be accessed at: http://www.mdpi.com/1996-1944/7/12/7670/s1.

## List of Symbols

**Parameter**	**Definition**
*a*	Stokes’ radius (nm)
*A*	Surface area of stent (*A* = 2π*r*^2^*h*; m^2^)
*A_p_*	Surface area of pores (m^2^)
*C_b_*	Solute concentration of bulk solution at the outer membrane surface (at *r_out_*), (kg/m^3^)
*C_b0_*	Concentration in bulk solution at *r_out_* at *t* = 0 s (mg/mL)
*C_m_*	Solute concentration at membrane interface (at *r_out_*), (mg/mL or kg/m^3^)
*C_m0_*	Concentration in membrane at *r_out_* at *t* = 0 s (mg/mL)
*D*	Free solution diffusivity, *i.e.*, Renkin–Curry diffusivity (m^2^/s)
*D_e_*	Effective diffusivity (m^2^/s)
*D*_empirical_	Empirical diffusivity (m^2^/s)
*D_m_*	Membrane diffusivity (m^2^/s)
*D*_numerical_	Numerically-computed or theoretical diffusivity (m^2^/s)
*I_b_*	Fluorescent intensity of bulk solution at outer membrane surface (a.u.)
*id*	Inner diameter (mm)
*I_m_*	Fluorescent intensity at membrane interface (at *r_out_*), (a.u)
*J*	Outward flux (kg/s)
*K*	Partition coefficient
*L*	Length (cm)
*MW*	Molecular weight
*od*	Outer diameter (mm)
*r*	Radial position (m)
*r_in_*	Inner radius of stent (m)
*r_out_*	Outer radius of stent (m)
*t*	Membrane tortuosity
*t*	Time (s)
*th*	Membrane thickness
*V_b_*	Volume of bulk solution (m^3^)
ω*_r_*	Hydrodynamic drag
ξ	Non-dimensionalized concentration
ξ*_b_*	Non-dimensionalized bulk concentration
ξ*_m_*	Non-dimensionalized concentration at membrane interface (at *r_out_*)
τ	Non-dimensionalized time
τ*_o_*	Membrane tortuosity
χ	Non-dimensionalized radial position
χ_1_	Non-dimensionalized inner radius
χ_2_	Non-dimensionalized outer radius
